# COVID-19 Induced Cardiomyopathy Successfully Treated with Tocilizumab

**DOI:** 10.1155/2022/9943937

**Published:** 2022-04-06

**Authors:** Ariyon Schreiber, Kalaimani Elango, Christoph Sossou, Sadaf Fakhra, Shabada Asad, Chowdhury Ahsan

**Affiliations:** ^1^Department of Cardiology-University of Nevada, Las Vegas-Kirk Kirkorian School of Medicine, USA; ^2^Department of Internal Medicine-University of Nevada, Las Vegas-Kirk Kirkorian School of Medicine, USA; ^3^Department of Infectious Disease-University of Nevada, Las Vegas-Kirk Kirkorian School of Medicine, USA

## Abstract

*Background*. Currently, the literature regarding the management of COVID-19 induced cardiomyopathy with reduced ejection fraction is limited. In this case report, we present the first documented case of COVID-19 induced myocardial stunning leading to severely reduced LV systolic function that was reversed by the administration of corticosteroids and tocilizumab. *Case Summary*. A 39-year-old female with well controlled systemic hypertension, tested positive for SARS-CoV-2 RNA and underwent self-isolation for 14 days. Patient presented to our facility a month later with one-week history of progressively worsening generalized body aches, chills, fever, watery diarrhea, nausea with associated mild dry nonproductive cough, shortness of breath and nonspecific chest pain. Initial labs demonstrated that she was COVID-19 positive, elevated troponin (4.295 ng/ml), and elevated BNP (2,291 pg/ml). Her initial Transthoracic echocardiography demonstrated an Left ventricular ejection fraction (LVEF) of 20-25% with apical akinesis. After administration of tocilizumab and corticosteroids, patient demonstrated interval improvement with LVEF improving to 50-55% within days. Her labs confirmed these findings with improved troponin (0.858 ng/ml) and BNP (209 pg/ml). *Discussion*. This case demonstrates that it can be safe and efficacious to use tocilizumab and corticosteroids in patients with COVID-19 induced cardiomyopathy. These finding suggest that cytokine storm is the predominant mechanism by which COVID-19 induced cardiomyopathy occurs. Additional studies are required to determine the role of corticosteroids and tocilizumab in management of this condition.

## 1. Introduction

It has been demonstrated that SARS-CoV-2 (COVID-19) can induce myocardial injury consequent to left ventricular (LV) systolic dysfunction; however, the exact pathophysiology is not fully understood [1]. A number of mechanisms have been proposed. The most commonly proposed mechanism is myocardial injury as a result of cytokine storm [2]. This is manifested by a parallel rise in inflammatory biomarkers and cardiac troponins alongside consequent left ventricular systolic dysfunction. Acute inflammation can trigger plaque destabilization, resulting in acute coronary syndrome as well as oxygen supply and demand mismatch-induced myocardial injury similar to type 1 and type 2 myocardial infarctions [1]. Another mechanism which has been hypothesized is direct invasion of the myocardium by the virus itself resulting in acute cardiomyopathy. However, data supporting this hypothesis are limited to case reports or case series, none of which have documented definitive diagnosis with endomyocardial biopsy as there is a paucity of inflammatory and cellular infiltrate as seen in myocarditis [2, 3]. Furthermore, autopsy series have failed to demonstrate myocardial necrosis as a result of COVID-19 [2]. Despite the fact that myocardial damage in the setting of COVID-19 infection is likely related to an exaggerated inflammatory response, direct myocardial invasion cannot be excluded.

Currently, the literature regarding management of COVID-19 induced cardiomyopathies is limited [4]. To that end, we present a case of COVID-19 induced cardiomyopathy that was reversed by the administration of corticosteroids, tocilizumab, and a number of other supportive medications.

## 2. Case Presentation

A 39-year-old female with well-controlled systemic hypertension tested positive for SARS-CoV-2 via PCR and underwent self-isolation for 10 days. She was admitted to our facility a month later with a three-day history of progressively worsening generalized body aches, chills, fever, watery diarrhea, nausea, mild dry nonproductive cough, mild shortness of breath, and nonspecific chest pain. She had minimal oxygen requirements saturating well above 90% on 2 L nasal cannula.

Her vital signs were significant for hypotension (blood pressure 82/44 mmHg), sinus tachycardia (heart rate of 132 beats/min), and axillary body temperature of 101.5°F (38.6°C). Cardiovascular exam demonstrated S3, 2+ pulses, warm extremities with bilateral pitting edema. Pulmonary exam was unremarkable, and no significant clinical symptoms of congestive heart failure or fluid overload were identified.

**Figure 1:**
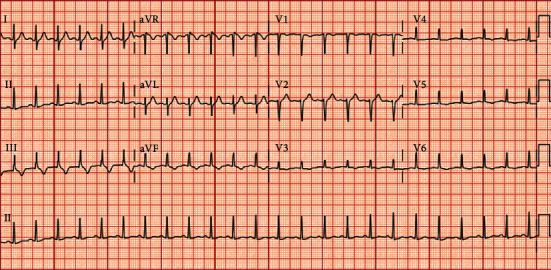
ECG from day of admission demonstrating sinus tachycardia without ST-T changes.

Electrocardiogram (ECG) showed sinus tachycardia (Figure 1). Laboratory studies demonstrated positive SARS-CoV-2 RNA PCR, troponin-I 4.295 ng/mL, and BNP 2291 pg/mL. Inflammatory markers for lactate dehydrogenase (LDH), ferritin, C-reactive protein, procalcitonin, and IL-6 were also elevated (Table 1). Chest X-ray and computed tomography angiography of the chest were unremarkable. Blood cultures were negative and no antibiotics were given throughout her admission.

**Figure 2:**
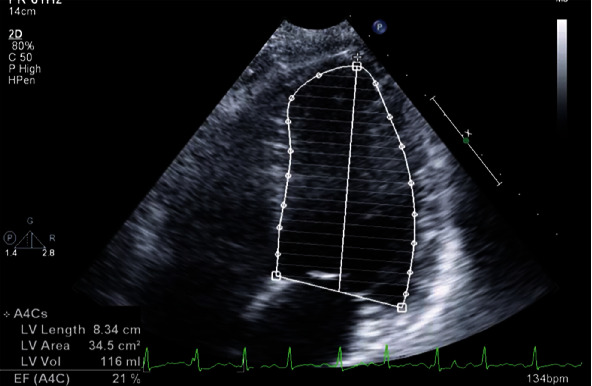
Simpson's method of disc's for measuring ejection fraction at the end of systole on admission.

**Figure 3:**
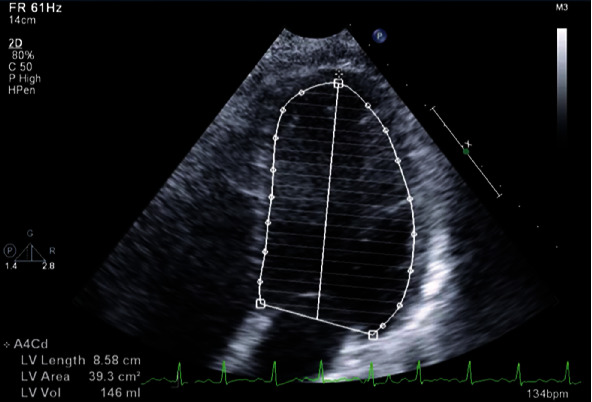
Simpson's method of disc's for measuring ejection fraction at the end of diastole on admission.

Cardiology was consulted regarding the patient's chest pain and elevated troponin. Transthoracic echocardiogram revealed left ventricular ejection fraction (LVEF) of 20-25% with LVIDd of 6.1 cm; apical akinesis but no pericardial effusion or significant valvular abnormalities (Figures 2 and 3).

After extensive discussion with our interdisciplinary team and the patient, she was started on tocilizumab 8 mg/kg once, dexamethasone 6 mg daily for 10 days, and furosemide 20 mg daily. She improved remarkably over a 72-hour period. She was subsequently started on guideline-directed medical therapy (GDMT) for systolic heart failure (i.e. losartan, carvedilol, and spironolactone).

**Figure 4:**
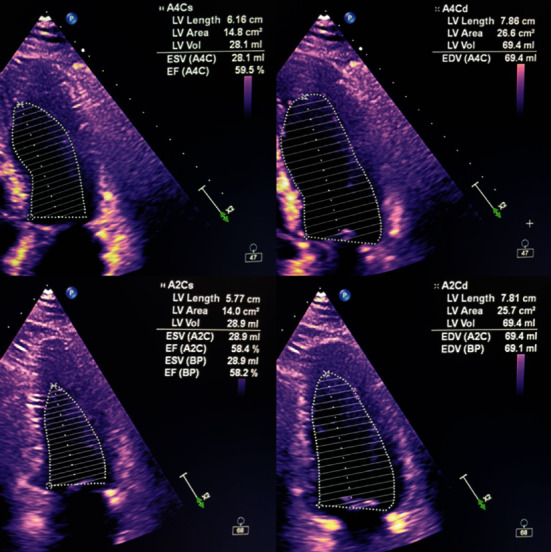
Simpson's method of disc's for measuring ejection fraction at end of systole and end of diastole on discharge demonstrating normal LVEF.

On hospital day seven, repeat transthoracic echocardiogram demonstrated interval improvement with LVEF to 50-55%, (Figures 4) and improvement in lab values (Table 1). The patient was successfully discharged home on hospital day 9. She was seen in cardiology outpatient clinic a week later and in medicine clinic 2 weeks afterward exhibiting excellent health.

## 3. Discussion

We present a case of COVID-19 associated cardiomyopathy most likely related to cytokine storm, Takotsubo stress induced cardiomyopathy (TC), or an acute coronary syndrome whereafter the patient showed complete recovery through treatment with tocilizumab and supportive vasoactive medications.

The differential for patients with COVID-19 and concomitant signs of acute coronary syndrome (ACS) includes a very broad spectrum of disease classified as COVID-19 associated acute cardiovascular syndrome [1]. Although our patient presented with mild chest pain and elevated troponin, coronary angiography was not pursued due to the low suspicion for occult plaque rupture or coronary arterial disease given her age, mild symptoms, and lack of signs of ischemia on ECG. Furthermore, a number of anecdotal reports have described cases of acute myocardial injury characterized by marked cardiac troponin elevation accompanied by ST-segment elevation or depression on ECG and angiography, often without coronary artery disease or culprit lesions identified on heart catheterization [5].

TC is another possible etiology. The most widely accepted hypothesis is thought to be related to complex systemic responses to acute severe stress and the response of the cardiovascular system to sudden surges in endogenous or exogenously-administered catecholamines [ 6]. In primary TC, acute cardiac symptoms resulting from emotional or physical stress are the main reason for seeking medical attention [6]. In contrast, secondary TC develops in patients who are hospitalized for other reasons [6]. The intense sympathetic stimulation in these patients caused by the primary condition or its treatment results in the development of TC [7, 8]. Some of the commonly identified triggers for the development of secondary TC are respiratory conditions, intubation, medication use, epinephrine use, anxiety and beta-blocker withdrawal [6, 9–11]. Many of these triggers are typically present in patients with COVID-19 pneumonia; however none of them were present in our patient [6]. Although her overall course appears consistent with a TC with temporary apical akinesia, no angiography was performed and without angiographic evidence of absence of obstruction we were unable to diagnose her with Takotsubo stress induced cardiomyopathy.

Due to the similarity of myocardial injury patterns in other cardiovascular pathologies, exact etiology is often difficult to determine and our case is no exception [1].Although CT coronary angiography, cardiac MRI, and endomyocardial biopsy were not performed due to material limitations, we suspect that her overall condition was related to sequalae from cytokine storm as no other causes of cardiomyopathy were identified.

As discussed, cytokine activation appears to be a prominent feature of severe COVID-19 illness with marked elevations of IL-6 along with other inflammatory markers [1]. Tocilizumab is an IL-6 inhibitor that has been FDA approved for chimeric antigen receptor (CAR) T cell-induced cytokine release syndrome (CRS) and has shown promise in the treatment of COVID-19 associated acute coronary syndrome [12, 13]. Several early small observational studies failed to show mortality benefit of tocilizumab in COVID-19 and expressed concern for secondary infections, increased transaminases, infusion reaction, and neutropenia [14–23]. However, more recent advancements from a well-designed multicenter study out of Oxford University using tocilizumab demonstrated shorter hospital stays and decreased need for mechanical ventilation [24].

To our knowledge, no current studies have been conducted to assess the efficacy of corticosteroid therapy on COVID-19 induced cardiomyopathy, specifically. Due to the lack of evidence regarding management for COVID-19 induced cardiomyopathy, we relied on evidence-based strategies employed to overcome cytokine storm caused by CAR therapy by increasing doses of corticosteroids [25].

We did not administer remdesivir or convalescent plasma due to the occurrence of symptoms more than a month beyond COVID-19 infection and quarantine; presentation of symptoms occurred after the viral phase which would thus make the use of antiviral therapy and convalescent plasma superfiluous [26]. Furthermore, despite the early promise for both of these treatments, the most recently published large trials have revealed no efficacy of either treatment against COVID-19 infection [27, 28].

This patient's prompt response to tocilizumab and corticosteroids appears to support the underlying hypothesis that cytokine storm is the primary mechanism by which COVID-19 induces cardiomyopathy and myocardial stunning. Presently, the exact pathologic mechanism of COVID-19 induced cardiomyopathy is unclear and evidence that COVID-19 invades the myocardium directly is limited to only a handful of case reports [2, 29–31].

## 4. Conclusion

SARS-CoV-2 continues to complicate the health of millions of people worldwide but guidelines for the management of COVID-19 associated cardiomyopathy have not yet been established. Nevertheless, we successfully employed treatment strategies for COVID-19 associated cardiomyopathy based on our understanding of the pathogenesis and from previous experience in treating COVID-19. Greater consistency and efficacy in its treatment will be undoubtedly uncovered from further successful investigation of the exact mechanism and appropriate management of COVID-19 associated cardiomyopathy. More data is required to determine the risks and benefits involved in using tocilizumab in the management of COVID-19 associated cardiomyopathy but the successful outcome of this case lends hope for a consensus and development of common guidelines for treatments of COVID-19 associated cardiomyopathy.

## Figures and Tables

**Table 1:** Demonstrating all lab values during her Hospital course.

	Reference range	Hospital day 1	Hospital day 2	Hospital day 3	Hospital day 5	Hospital day 7	Hospital day 9
Troponin I (NG/ML)	0.020-0.040	4.295	4.294	0.858			
Brain Natiuretic peptide (pg/ml)	<99		2,291(H)			209(H)	
Lactate dehydrogenase (U/L)	125-243		229	294 (H)	229	330 (H)	208
C-reactive protein(mg/L)	<3.0		>200(H)	>200(H)	71.3(H)	37.7(H)	23.2(H)
D-dimer (MG/L)	<0.49		1.80 (H)	1.56(H)	0.96(H)	0.95(H)	1.25(H)
Ferritin (NG/ML)	7.0-271.0		408.6(H)	588.9(H)	304.8(H)	255.4	223.1
Interleukin-6 (pg/ml)	0.00-12.2		46.5 (H)				

## Data Availability

All data is included in the Case report.
